# A Case of West Nile Virus Neuroinvasive Disease Presenting With Isolated Diplopia

**DOI:** 10.7759/cureus.65777

**Published:** 2024-07-30

**Authors:** Elizabeth A Olsen, Alexander K Quinones, Timothy L Vo

**Affiliations:** 1 Emergency Medicine, Rocky Vista University College of Osteopathic Medicine, Parker, USA; 2 Emergency Medicine, University of Colorado Anschutz Medical Campus, Aurora, USA

**Keywords:** viral meningoencephalitis, viral meningitis, infectious encephalitis, aseptic encephalitis, west nile virus infection, west nile virus encephalopathy, neuroinvasive west nile virus

## Abstract

West Nile virus (WNV) is a single-stranded RNA virus causing a wide spectrum of diseases. Neuroinvasive conditions such as meningitis and encephalitis are feared complications of WNV infection. Here, we describe the case of a 78-year-old male whose only initial presenting symptoms were fever and transient diplopia, whose initial MRI imaging with and without contrast did not reveal any abnormalities. He was discharged, only to return to care the next day; lumbar puncture was performed suggesting bacterial meningitis, and he was admitted and given antibiotics. Repeat MRI was negative, and he developed an altered mental status requiring intubation. WNV neuroinvasive disease was subsequently found after serology was performed. Supportive care was given, and he made a full recovery with no residual deficits. This case highlights an unusual presentation of WNV encephalitis and highlights the difficulty that can be present in diagnosing this disease.

## Introduction

West Nile virus (WNV) is a single-stranded, positive-sense RNA virus of the Flaviviridae family causing diseases ranging from subclinical infection to fulminant encephalitis ​[1]​. The virus is primarily transmitted through mosquito bites from the *Culex *species, *C tarsalis* and *C pipiens* ​[1]​. However, it can also be transmitted via transfusion of infected blood products, organ transplantation from an infected donor, percutaneously through occupational exposure, or mother to child via breastfeeding ​[2]​. WNV typically affects individuals living in or visiting endemic areas, particularly during outbreaks. It commonly affects those who spend time outdoors, especially between dusk and dawn. This can include individuals who have outdoor occupations or those who participate in outdoor activities ​[3]​. 

WNV typically has a subclinical presentation, and approximately 80% of patients contracting the virus will be asymptomatic ​[4]​. Most symptomatic cases will present as a self-limited febrile illness two to 14 days after an initial mosquito bite or other exposure, with associated headache, myalgias, arthralgia, nausea, and vomiting [5]. Symptoms are most commonly present in a three- to 10-day range but in some cases can persist for several weeks [5]. IgM is typically detectable three to eight days after the onset of viremia and often persists for over two months following the resolution of illness [6]​. Diagnosis of WNV is typically done via detection of serum IgM using enzyme-linked immunosorbent assay (ELISA) techniques [7]​. 

Less than one percent of infected individuals will progress to neuroinvasive WNV, which can present as meningitis, encephalitis, or flaccid paralysis [4]​. There are several risk factors that increase the likelihood of individuals developing neuroinvasive WNV, including increasing age, history of hypertension, diabetes mellitus, chronic renal disease, and compromised immune system ​[8]​. Patients with neuroinvasive disease can present with a range of symptoms, including high fever, headache, neck stiffness, stupor, disorientation, seizures, paralysis, and decreased consciousness ​[9]​. Some patients develop an acute flaccid paralysis characterized by asymmetric limb weakness and areflexia or hyporeflexia which can resemble poliomyelitis ​[10]​. Herein, we describe an unusual presentation of WNV encephalitis, with isolated fever and diplopia on initial presentation.

## Case presentation

A 78-year-old male presented to an academic emergency department in metro Denver, Colorado with fever and confusion. He had an onset of fever and transient diplopia the previous night and had presented to an outside community hospital ED, where computed tomography angiography (CTA) and MRI brain with and without contrast were performed. These were read as negative for the acute process, and he was discharged.

The next day, he was noted to have an onset of confusion and continued fever, and he presented to our ED. His diplopia had resolved. He was an avid golfer and had a high functional status at baseline, and he had not traveled out of state in the last several weeks. Further history from the patient himself was difficult due to his underlying level of confusion. His past medical history was notable for hyperlipidemia, type II non-insulin dependent diabetes, benign prostatic hypertrophy, hiatal hernia, and hypothyroidism. Vital signs were notable for a temperature of 39.1 degrees Celsius. On exam, he was somnolent with incomprehensible speech but was following commands in all four limbs and crossing the midline. He had no rash. His pupils were noted to be symmetrical, round, and reactive, and extraocular movements were noted to be symmetrical. His neck was noted to be supple without rigidity. No tremor or rigidity was noted.

A basic metabolic panel, complete blood count, troponin I, brain natriuretic peptide, respiratory viral panel, and urinalysis were performed and were normal; lactate was 2.3 mmol/L, and white blood cell count was 12.0 10^9/L (Table 1). A CT angiogram of the head and neck, a CT angiogram of the chest, and a CT of the abdomen and pelvis with contrast were performed showing no significant acute abnormality. MRI of the brain without contrast was repeated and was normal. A lumbar puncture was performed and CSF studies were obtained (Table 2), showing 560 white blood cells with 57% neutrophils, seven red blood cells, glucose of 76 mg/dL, and protein of 145 mg/dL. Initial gram stain was negative. On pathologist review, the CSF was noted to show circulating plasma cells with increased pleomorphism in addition to circulating inflammatory cells.

**Table 1:** Laboratory values

Parameter	Patient value	Normal value range
Sodium	135	134-145 mmol/L
Potassium	4.5	3.5-5.1 mmol/L
Chloride	102	98-109 mmol/L
Carbon dioxide	20	22-30 mmol/L
Glucose	149	70-105 mg/dL
Blood urea nitrogen	20	9-20 mg/dL
Creatinine	1.08	0.66-1.25 mg/dL
Calcium	8.7	8.4-10.2 mg/dL
Lactate	2.3	0.5-2.0 mmol/L
White blood cells	12.0	4.0-11.1 10^9/L
Hemoglobin	13.6	14.3-18.1 g/dL
Hematocrit	42.6	39.2-50.2%
Platelets	218	150-400 10^9/L
Neutrophil absolute	10.4	1.8-6.6 10^9/L
Lymphocyte absolute	0.8	1.0-4.8 10^9/L
Monocyte absolute	0.8	0.2-0.9 10^9/L
Eosinophil absolute	0.0	0.0-0.4 10^9/L
Basophil absolute	0.0	0.0-0.2 10^9/L
Immature granulocyte absolute	0.0	0.0-0.1 10^9/L

**Table 2:** Cerebrospinal fluid (CSF) analysis

Parameter	Patient value	Normal value range
Color	Colorless	
Appearance	Clear	
CSF tube number	#4	
Nucleated cell count	560	0-5 10^6/L
CSF red blood cell count	7	0 10^6/L
Segmented neutrophil CSF	57%	0-6%
Lymphocyte CSF	15%	40-80%
Monocyte/macrophage CSF	28%	15-45%
Glucose CSF	76	40-70 mg/dL
Protein total	145	12-60 mg/dL

Given the neutrophilic predominance of his CSF studies, the patient was initially admitted to the floor with a working diagnosis of bacterial meningitis, and IV vancomycin, ceftriaxone, ampicillin, acyclovir, and dexamethasone were started. Viral serologies including herpes simplex, varicella zoster, and WNV were added to the available CSF. EEG was performed showing theta/delta slowing, suggestive of non-specific cerebral dysfunction. On hospital day two, he developed increasing somnolence and his respiratory status declined. He was intubated for airway protection and hypoxemia and was transferred to the intensive care unit.

WNV serologies returned positive: IgM was positive at 4.32 IV and IgG was negative at 0.16 IV. Blood cultures did not demonstrate any growth. Antibiotic and antiviral therapies were discontinued. Supportive measures were continued. He was extubated on hospital day seven, was transferred back to the floor, and was able to be discharged to rehab on hospital day 14.

He followed up in the neurology clinic one month after discharge, where he reported persistent fatigue and orthostatic lightheadedness. Exam at that time was remarkable for 4/5 motor strength in the bilateral hip flexors, and the right lower extremity knee flexion, ankle dorsiflexion, and great toe extension; motor strength was noted to be 5/5 in the remainder of the limbs. Achilles reflex was noted to be absent bilaterally. Sensory and cranial nerve exam was noted to be normal. An MRI lumbar spine was ordered, which the patient did not schedule. He was last seen in a follow-up six months later in the neurology clinic, where he noted near full resolution of symptoms; motor exam at that time was noted to be normal.

## Discussion

Neuroinvasive WNV should be considered in any patients who present with aseptic meningitis or encephalitis, particularly during mosquito season in endemic areas. CT imaging will typically show no acute disease process. MRI may show signal abnormalities in the thalamus, brainstem, or spinal cord but is often normal until several weeks after onset of illness ​[9]​. Notably, in our patient, neither of his two MRIs displayed any CNS abnormalities.

Lumbar puncture with CSF analysis is key to the diagnosis of neuroinvasive WNV. Findings consistent with such include positive IgM antibodies against WNV, elevated protein, and moderate pleocytosis with a predominance of lymphocytes, although there may be elevated neutrophils early in infection [11]. On cytology of the CSF, plasmacytoid lymphocytes (seen in our patient) or large monocytic cells are often seen ​[12]​. Other differentials that should be considered with patients presenting similarly include other viral meningitis or encephalitis such as that due to HSV, bacterial meningitis due to listeria, mycobacterium tuberculosis, etc., and other demyelinating diseases. MRI, history of presenting illness, and CSF studies and culture are useful to differentiate these disease processes. 

Treatment of neuroinvasive WNV is primarily supportive, though some specific treatments have been investigated for potential benefits [13]​. Corticosteroids, while used, are controversial. Individual case reports have shown possible clinical improvement; however, a non-randomized study of 18 patients with neuroinvasive WNV showed no change in the duration of hospital stay in those who received corticosteroids when compared to those who did not [14]​. Although IVIG has theoretical benefits in patients with humoral deficiencies, a randomized control trial published in 2019 failed to show any benefit ​[15]​. Antivirals have not been proven efficacious either. Ribavirin showed possible harm, and acyclovir showed no change in the length of hospital stay or clinical benefit when compared to supportive treatment ​[16,17]​. 

The fatality rate of patients who develop neuroinvasive WNV is variable and is based on the severity of presentation. Presentation with meningitis has a fatality rate of about two percent, while presentation with encephalitis has a fatality rate of 14%, and presentation with acute flaccid paralysis has a fatality rate of approximately 13% ​[18]​. Both increased age and coma at the time of patient presentation are independent predictors of mortality ​[19]​. One study found that approximately 37% of patients will fully recover from the disease after one year, while other patients may have persistent muscle weakness, confusion, and/or lightheadedness. Some patients may develop long-term functional disabilities, cognitive impairment, and memory loss from the disease process ​[20]​.

## Conclusions

The diagnosis of neuroinvasive WNV is challenging; thus, healthcare providers should have a heightened awareness of this disease in their differential diagnosis in patients exhibiting symptoms of aseptic meningitis, encephalitis, or acute flaccid paralysis. This case underscores the importance of considering neuroinvasive WNV in patients presenting with fever and neurological symptoms, particularly in endemic areas. Our patient’s initial presenting symptoms were fever and isolated diplopia only. Accurate diagnosis relies on CSF analysis and serology, as imaging may not show abnormalities in the early stages, as this case highlights. Supportive care remains the mainstay of treatment, with no specific antiviral therapy proven effective. Awareness and timely recognition of this condition are crucial for managing symptoms and improving patient outcomes.
